# USP9X trims STAT1 to curb oncostatin M activity and intestinal inflammation

**DOI:** 10.1038/s41423-026-01445-4

**Published:** 2026-06-25

**Authors:** Ahmed N. Hegazy

**Affiliations:** 1 Charité - Universitätsmedizin Berlin, corporate member of Freie Universität Berlin and Humboldt-Universität zu Berlin, Department of Gastroenterology, Infectious Diseases and Rheumatology, Berlin, Germany; 2 Deutsches Rheuma-Forschungszentrum, a Leibniz Institute, Berlin, Germany; 3 Cluster of Excellence ImmunoPreCept, Charité - Universitätsmedizin Berlin, Berlin, Germany

**Keywords:** Translational immunology, Oncostatin M

**Fig. 1:**
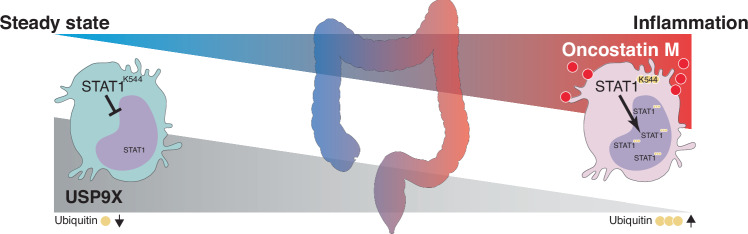
USP9X–STAT1–OSM axis in intestinal macrophages. In USP9X-sufficient macrophages (left), USP9X removes K27-linked ubiquitin chains from STAT1 lysine 544, limiting STAT1 nuclear translocation and OSM transcription. In USP9X-deficient macrophages (right), K27-linked chains accumulate at K544, enhancing STAT1 nuclear import. STAT1 occupies the OSM promoter and drives OSM expression, promoting proinflammatory myeloid accumulation and colitis

Macrophages are critical regulators of intestinal homeostasis. They sense their environment rapidly and adapt their function accordingly. In IBD, proinflammatory macrophages accumulate in the mucosa, sustain cytokine production, and drive repeated cycles of tissue damage. Oncostatin M (OSM) is a key cytokine involved in intestinal inflammation [1, 2]. Produced primarily by myeloid cells and T cells in inflamed mucosal tissue, OSM signals through stromal and epithelial cells to perpetuate inflammation and resist resolution, and its mucosal expression predicts the failure of anti-TNF therapy [1, 3]. Despite its clinical importance, what controls OSM production in the inflamed gut remains poorly understood. A new study by Zhang and colleagues, published in this issue, addresses that question [4]. The authors identify USP9X, a deubiquitinase, as a critical brake on macrophage OSM expression. USP9X removes K27-linked ubiquitin chains from STAT1 at lysine 544, limiting STAT1 nuclear entry and OSM transcription. The loss of this checkpoint in myeloid cells worsens experimental colitis, and neutralizing OSM rescues it. This work defines a USP9X–STAT1–OSM axis that connects ubiquitin editing to a cytokine with direct clinical relevance in IBD (Fig. 1).

Adaptation to environmental cues is central to macrophage function in intestinal inflammation. The transcriptional and metabolic programs underlying this process are increasingly well characterized, but posttranslational regulation remains poorly understood. Ubiquitination is among the most versatile posttranslational modifications in immune cells. It controls protein stability, signaling scaffold assembly, and transcription factor activity through a code embedded in ubiquitin chain topology. K48-linked chains target proteins for proteasomal degradation. K63-linked chains coordinate signaling. K27-linked chains are less studied but have recently emerged as regulators of nuclear localization in innate immunity [5]. Several deubiquitinases (DUBs), including JOSD2, USP7, and OTUD6A, are known to regulate macrophage function in the inflamed gut [6–8]. USP9X now joins this list, with a distinct substrate and a distinct chain topology.

By showing that USP9X transcript levels are reduced in mucosal biopsies from IBD patients across multiple datasets, Zhang et al. established the clinical relevance of USP9X, and its expression inversely correlated with the Mayo endoscopic score and erythrocyte sedimentation rate. Mining available single-cell RNA sequencing localizes this reduction to the monocyte/macrophage compartment, specifically to immature monocytes that coexpress elevated levels of TNF-α and IL-6. Immunofluorescence staining of colonic biopsies confirmed that USP9X protein expression was reduced in CD68^+^ macrophages from UC patients. These correlative observations set up the mechanistic work that follows.

Functional significance is then established through gain- and loss-of-function models. Myeloid-specific deletion of USP9X worsened colitis in both the DSS-induced and TNBS-induced models, with increased inflammatory infiltration, greater weight loss, shorter colons, and elevated serum cytokines. Macrophage depletion with clodronate abolished the difference between genotypes, confirming that the phenotype was macrophage dependent. In contrast, intraosseous delivery of an AAV9 vector system resulted in myeloid-specific USP9X overexpression and protection against DSS-induced colitis.

The molecular mechanism is identified through an integrated proteomics approach. Label-free ubiquitylomics and anti-FLAG immunoprecipitation mass spectrometry converge on STAT1 as a direct USP9X substrate, with lysine 544 as the modified residue. Using a panel of ubiquitin mutants that permit only a single linkage type, the authors show that K27-linked chains specifically accumulate on STAT1 and are removed by USP9X. A K544R point mutation abolishes both the modification and USP9X deubiquitinase activity at this site. Importantly, K27 ubiquitination at K544 accelerates and amplifies STAT1 nuclear import. In USP9X-deficient macrophages, STAT1 nuclear accumulation peaks earlier and increases after LPS and IFN-γ stimulation. CUT&Tag sequencing of lamina propria macrophages from knockout mice revealed broadly increased STAT1 chromatin occupancy, with OSM emerging as the top inflammatory gene at the intersection of increased STAT1 promoter binding and transcriptional upregulation. ChIP‒qPCR confirmed elevated STAT1 occupancy across three regions of the OSM promoter. The STAT1 K544R mutation failed to induce OSM upon USP9X knockdown, confirming that this site is functionally needed. OSM-neutralizing antibody treatment completely abolished the difference in colitis severity between USP9X-deficient and control mice and restored normal monocyte-to-macrophage maturation.

In this study, STAT1 was established as a direct transcriptional activator of OSM in intestinal macrophages. Prior work identified regulatory elements in the OSM promoter, but the upstream driver in macrophages had remained unidentified. It also adds K27-linked ubiquitination to the growing list of posttranslational modifications that tune STAT1 nuclear dynamics, in addition to methylation, SUMOylation, and linear ubiquitin chains [9, 10]. Unlike those modifications, K27-Ub at K544 does not broadly activate interferon-stimulated genes. It preferentially induces OSM, which likely explains why USP9X deficiency drives gut inflammation without triggering a systemic interferon response.

Several questions remain unanswered. In human IBD, USP9X expression is reduced but not absent; however, the mechanistic studies here rely on the complete deletion of USP9X in myeloid cells. Whether the partial loss seen in patients is sufficient to meaningfully disrupt the USP9X–STAT1–OSM axis or whether disease progression reflects a gradual erosion of this checkpoint over time remains unclear. The E3 ligase that promotes K27-linked ubiquitination of STAT1 K544 is also unidentified. IFN-γ and LPS reduce USP9X protein expression in macrophages, but the downstream molecular signals driving its reduction in myeloid cells have not been established. How USP9X expression fluctuates across the disease course between flare-up and remission remains unclear. Whether advanced therapies, especially anti-TNF agents and JAK inhibitors, modulate USP9X expression is important for understanding how drug-specific mechanisms promote inflammation resolution. Finally, whether monocyte-to-macrophage maturation blockade reflects a cell-intrinsic consequence of USP9X loss or is driven indirectly by OSM through STAT3 signaling remains to be determined.

In addition to the gut, OSM is elevated in rheumatoid arthritis, asthma, and COVID-19-associated lung injury. Whether the USP9X–STAT1 axis controls macrophage OSM expression in those settings is an interesting question. The gut may be where this axis was found, but it is unlikely to be where it ends.
